# Response and survival of dogs with proteinuria (UPC > 2.0) treated with angiotensin converting enzyme inhibitors

**DOI:** 10.1111/jvim.16864

**Published:** 2023-10-10

**Authors:** Emily A. Fulton, Alix R. McBrearty, Darren J. Shaw, Alison E. Ridyard

**Affiliations:** ^1^ The University of Glasgow Small Animal Hospital, School of Biodiversity, One Health and Veterinary Medicine, 464 Bearsden Road Glasgow G61 1QH United Kingdom; ^2^ VetsNow Hospital Glasgow, 123‐145 North Street Glasgow G3 7DA United Kingdom; ^3^ Royal (Dick) School of Veterinary Studies, The University of Edinburgh, Easter Bush Campus Roslin EH25 9RG United Kingdom

**Keywords:** benazepril, enalapril, glomerular, urine protein creatinine ratio (UPC)

## Abstract

**Background:**

Angiotensin‐converting enzyme inhibitors (ACEi) are a recommended treatment for glomerular proteinuria. Frequency of response to ACEi and the association of achieving proposed urine protein‐to‐creatinine ratio (UPC) targets on survival is unknown.

**Objectives:**

To determine response rates to ACEi therapy and whether a positive response is associated with improved survival.

**Animals:**

Eighty‐five dogs with proteinuria (UPC > 2.0).

**Methods:**

Retrospective study including dogs (UPC > 2.0) prescribed an ACEi for treatment of proteinuria. Baseline creatinine, albumin, cholesterol, UPC, and systolic blood pressure were recorded, and cases reviewed to track UPC. Treatment response was defined as achieving a UPC of <0.5 or reduction of ≥50% from baseline within 3 months. Outcome data were collected to determine overall and 12‐month survival.

**Results:**

Thirty‐five (41%) dogs responded to ACEi treatment. Treatment response was statistically associated with both median survival time (664 days [95% confidence interval (CI): 459‐869] for responders compared to 177 [95% CI: 131‐223] for non‐responders) and 12‐month survival (79% responders alive compared to 28% non‐responders). Baseline azotemia or hypoalbuminemia were also associated with a worse prognosis, with odds ratios of death at 12 months of 5.34 (CI: 1.85‐17.32) and 4.51 (CI: 1.66‐13.14), respectively. In the 25 dogs with normal baseline creatinine and albumin, response to treatment was associated with 12‐month survival (92% responders alive compared to 54% non‐responders, *P* = .04).

**Conclusions and Clinical Importance:**

When the UPC is >2.0, achieving recommended UPC targets within 3 months appears to be associated with a significant survival benefit. Response to treatment is still associated with survival benefit in dogs with less severe disease (no azotemia or hypoalbuminemia).

**Abbreviations**
ACE: angiotensin converting enzyme
ACEi: angiotensin converting enzyme inhibitor
ARB: angiotensin receptor blockers
CKD: chronic kidney disease
MST: median survival time
PLN: protein losing nephropathy
RAAS: renin‐angiotensin‐aldosterone system
SBP: systolic blood pressure
UPC: urine protein‐creatinine ratio

## 1 INTRODUCTION

Proteinuria is a hallmark of glomerular disease and when the urine protein‐creatinine ratio (UPC) is >2.0 in the absence of pre‐ and post‐renal causes, glomerular disease is often present.^1^ While renal biopsy is required for a definitive pathological diagnosis thereby informing treatment choices, it is infrequently performed, and when the UPC is >2.0, a presumptive diagnosis of glomerular proteinuria is usually reached by noninvasive exclusion of pre‐ or post‐renal causes of proteinuria.

Glomerulopathies are a major cause of chronic kidney disease (CKD) and eventual renal failure in dogs,^2^ with proteinuria per se likely contributing to progressive renal disease.^3^, ^4^, ^5^ Even in the absence of a histopathological diagnosis, management of cases with presumed glomerular proteinuria is aimed at reduction of proteinuria and management of clinically relevant complications.^6^, ^7^

The 2013 ACVIM Consensus statement for standard therapy of glomerular disease in dogs states modification of the renin‐angiotensin‐aldosterone system (RAAS) should be standard of care for dogs with glomerular proteinuria and this is often achieved by use of an angiotensin converting enzyme inhibitor (ACEi).^7^ Benazepril and enalapril are commonly used ACEi. The proposed target of ACEi therapy is either a reduction in UPC to <0.5 or a >50% reduction from baseline.^7^ However, there is currently limited data on how frequently dogs treated with ACEi meet such treatment targets and whether achieving these targets improves disease progression and overall survival.

The aims of this retrospective study were to determine the proportion of dogs with a UPC > 2.0 achieving the proposed target reduction in UPC within 3 months of starting an ACEi and determine whether a positive response to treatment was associated with improved survival. To investigate for possible confounding factors, secondary aims were to evaluate whether clinicopathological markers of disease severity in the 30‐days before treatment commencement had an association with either response to treatment or survival.

It was hypothesized that response to ACEi therapy within 3 months would be associated with improved 12‐month survival.

## 2 MATERIALS AND METHODS

Clinical records of dogs referred to a University Teaching Hospital were searched to identify those with a UPC > 2.0 prescribed an ACEi for treatment of proteinuria between January 2006 and April 2021. Dogs were excluded if they were prescribed ACEi for any other condition; were receiving concurrent therapy known to affect renal protein loss (tyrosine kinase inhibitors, corticosteroids, or angiotensin receptor blockers [ARBs]) at the time of baseline urinalysis or did not have a baseline urinalysis and biochemistry performed within 30‐days before starting ACEi therapy. UPC was measured as standard at the laboratory performing urinalysis. Dogs with incomplete records were not included. Additionally, those without at least 1 follow‐up urinalysis (including UPC measurement) at the university laboratory within 3 months of starting ACEi therapy were excluded. Screening for infectious diseases was not required for inclusion as the prevalence of infectious diseases that can contribute to proteinuria is low in the UK. Cases with an active sediment on urinalysis or co‐morbidities were not excluded.

Medical records were reviewed and signalment and baseline variables recorded. Baseline variables were defined as the creatinine, albumin, cholesterol, UPC, and systolic blood pressure (SBP) values obtained within 30 days before ACEi commencement. If several values were available within this time frame, the ones closest to the start of ACEi treatment were used. The choice and starting dose of ACEi (mg/kg/day), concurrent comorbidities and diagnoses were recorded, as were medications, dietary management and omega‐3 fatty acid supplementation already being administered or started at the time of starting ACEi therapy. For clinical threshold analysis, azotemia was defined as a creatinine >1.4 mg/dL (>125 μmol/L); hypoalbuminemia an albumin <2.5 g/dL (<25 g/L) and hypercholesterolemia as cholesterol >348 mg/dL (>9 mmol/L).^8^, ^9^ Severe proteinuria was defined as a UPC > 3.5.^10^ Using the ACVIM Consensus Guidelines for Systemic Hypertension, normotensive or pre‐hypertensive dogs were classed as “non‐hypertensive” (SBP < 159 mm Hg) while those with hypertension or severe hypertension were classed as “hypertensive” (SBP ≥ 160 mm Hg).^11^ The following data were collected from subsequent re‐checks: date, UPC, new dose of ACEi if changed and whether ARBs or corticosteroids were started.

Dogs were categorized as responders or non‐responders. Treatment response was defined as a reduction of UPC to ≥50% from baseline or to <0.5^7^ at any follow‐up visit within 3 months of starting ACEi. If dogs did not achieve these targets by 3 months, they were classed as non‐responders. Dogs were also classed as non‐responders if they were started on an ARB or corticosteroid within 3 months and before the UPC targets being achieved or if ACEi therapy was withdrawn because of progressive azotemia or hyperkalemia at any time during the first 3 months before the UPC targets being achieved.

To perform survival analyses, the date of death/euthanasia, or the point at which the patient was lost to follow‐up (last known to be alive) was recorded. If available, the reason for death/euthanasia was recorded. Dogs were then additionally classified based on their status 12 months after initiation of ACEi as either alive, dead, or lost to follow‐up.

### 2.1 Statistical analysis

Normality testing (using the Anderson‐Darling method) indicated that for 3 of the 4 continuous variables (creatinine, cholesterol, UPC) non‐parametric statistical analyses were required, therefore, for descriptive statistics the median and range were reported. Pearson chi‐squared analysis was employed to determine the association between baseline clinical threshold variables (baseline azotemia, hypoalbuminemia, hypercholesterolemia, severe proteinuria, and hypertension) and treatment response. Baseline variables were also compared between dogs that did and did not respond to treatment utilizing either Mann‐Whitney *U* or Pearson chi‐squared tests as appropriate.

Overall survival data (using all‐cause mortality) were assessed using Kaplan Meier analysis with the Log‐Rank (Mantel‐Cox) test used to assess for a difference in median survival time (MST) between responder and nonresponder dogs. The association between the presence of baseline azotemia, hypoalbuminemia, hypercholesterolemia, severe proteinuria, hypertension, and overall survival were assessed similarly.

Pearson chi‐squared analysis was used to assess for a relationship between response to treatment and survival at 12 months. The associations between baseline creatinine, albumin, cholesterol and UPC and 12‐month survival were assessed using clinical thresholds and on a continuous basis while SBP was assessed on a clinical threshold basis only. For the clinical threshold analysis, Pearson chi‐squared tests were again employed, and Mann‐Whitney tests were used to investigate the continuous data.

Univariate logistic regression was carried out to generate odds ratios (OR) and 95% confidence intervals (CI) of survival to 12 months for response to treatment and baseline parameters. Parameters with a *P* value <.2 were eligible for inclusion into the multivariable analysis. Parameters fitting this criterion were entered into a multivariable logistic regression model and terms removed until a minimum model was obtained with only statistically significant ORs remaining.

To further account for possible interplay between baseline values of variables and treatment response on 12‐month survival, a categorical tree was generated. This analytical technique was chosen because of the unbalanced data set with potentially different combinations of baseline clinical threshold values present in different dogs.^12^ The categorical tree aimed to assess for the impact of numerous variables (treatment response, baseline azotemia/hypoalbuminemia/hypercholesterolemia or severe proteinuria) on 12‐month survival and rank them in order of importance as well as identify combinations of factors leading to the highest risk of death/greatest chance of survival. The categorical tree analysis allowed for binary division of data between groups of categories; the category that led to the biggest difference regarding 12‐month survival represented the first division. One subset was then considered, and the model then assessed which category led to the biggest difference in 12‐month survival in that sub‐group of dogs. The different “branches” of the tree were independent of each other in terms of what binary partitions were presented. This binary partitioning was continued for smaller and smaller subsets of data until no differentiation in terms of prevalence was possible.

Statistical analysis was performed using commercially available software SPSS (IBM SPSS Statistics 27) and R (v4.2.1© 2021, The R Foundation for Statistical Computing). Statistical significance was taken as *P* < .05.

## 3 RESULTS

### 3.1 Case selection

The initial database search identified 1000 dogs with a UPC > 2.0. Of these, 245 were prescribed an ACEi. Thirty‐one dogs were not prescribed an ACEi for treatment of proteinuria; these dogs were excluded. Of the remaining 214 dogs, 24 were excluded as they were receiving concurrent medication known to affect UPC, 12 dogs were excluded as their baseline biochemistry or urinalysis was performed >30 days before starting the ACEi. A further 93 were excluded because of incomplete clinical records or lack of follow‐up within 3 months. Therefore, 85 dogs were included.

### 3.2 Study cohort characteristics

Of the 85 dogs, 58% were female (n = 49/85; neutered n = 34), and 42% were male (n = 36/85; neutered n = 15). The median age at the time of starting ACEi therapy was 8.70 years (range, 0.65‐13.85 years). Weight was available for 73 dogs; the median weight was 14.70 kg (range, 2.30‐50.0 kg). There were 11 crossbreeds with the remainder of the study cohort being purebreds.

At the time of starting ACEi, protein losing nephropathy (PLN) was listed as the diagnosis for 57% (n = 48/85) dogs. Thirteen dogs (15%) had renal diseases other than PLN listed as their diagnosis, 18% (n = 15/85) had at least 1 endocrinopathy while 12% (n = 10/85) had a diagnosis of neoplasia (of which 2 had concurrent endocrine disease). One dog was listed as having a type III hypersensitivity reaction.

Of the 15 dogs with endocrinopathies, 8 had hyperadrenocorticism of which 5 were receiving trilostane treatment before ACEi commencement and 2 were later started on trilostane. All 6 dogs with diabetes mellitus were receiving insulin therapy. One dog had hypothyroidism and was receiving levothyroxine before starting ACEi treatment. One dog was positive on serology for *Borrelia burgdorferi*, no other infectious diseases were documented. No dog underwent renal biopsy.

The median baseline creatinine was 1.14 mg/dL (range, 0.51‐6.30); 40.0% of dogs (n = 34/85) were azotemic.

The median baseline UPC was 6.61 (range, 2.15‐30.5), 80.0% of dogs had severe proteinuria (n = 68/85). The median baseline albumin was 2.5 g/dL (range, 0.8‐3.7 g/dL); 45.9% (n = 39/85) of dogs were hypoalbuminemic. The median cholesterol was 331.5 mg/dL (range, 83.8‐1409.8); 44.7% (37/84) of dogs were hypercholesterolemic. Baseline SBP measurement was available for 46 dogs; 52.2% (n = 24) of these were classed as being hypertensive (hypertension n = 10, severe hypertension n = 14) before starting ACEi therapy.

Six dogs were prescribed enalapril while the remainder received benazepril. The starting dose of ACEi was available for 73 dogs with a median of 0.5 mg/kg/day (range, 0.16‐1.52 mg/kg/day). At the time of starting ACEi therapy 8% (n = 7/85) of dogs were already receiving a renal diet and a further 29% (n = 25/85) of dogs were started on a renal prescription diet alongside ACEi. None of the dogs were on omega‐3 fatty acid supplementation before starting an ACEi, however, these were started in 9.4% (n = 8/85) of dogs at the same time as the introduction of ACEi, of these, 3 were concurrently started on a renal diet. Three dogs were already on anti‐thrombotic medication at the time of starting ACEi; a further 38% (n = 32/85) were started on such medications at the same time as starting ACEi. At the time of ACEi commencement, 8% (n = 7/85) were receiving amlodipine therapy, 3 of which had documented hypertension at baseline.

### 3.3 Response to treatment

Table 1 shows the baseline variables of dogs that were classed as responders and non‐responders. Statistically significant differences in baseline variables were not found between responders and non‐responders. Thirty‐five dogs (41%) responded to ACEi therapy within 3 months with only 1 achieving a UPC < 0.5. The median number of rechecks performed within 3 months in this group was 2 (range, 1‐5). The median time to response was 33 days (range, 3‐82 days) and the median dose at the time of response was 0.52 mg/kg/day (range, 0.19‐1.32 mg/kg/day). Of the dogs that responded, 10 had a further urine sample available within the 3‐month period after the 1 documenting a treatment response. Of these, 30% (n = 3/10) had a subsequent UPC that would not have satisfied criteria for successful response to treatment.

**TABLE 1:** Baseline variables of 85 dogs with a UPC > 2.0 that responded and did not respond to angiotensin converting enzyme inhibitors.

Variables	Responders (n = 35)	Non‐responders (n = 50)	*P*‐value
Age (years)	9.10 (0.65‐13.85)	8.50 (1.49‐10.11)	*P*_M_ = .95
Sex			
Female [neutered, %]	23 (65.7%) [14, 60.9%]	26 (52.0%) [20, 76.9%]	Sex: *P*_*χ*_ = .3 Neutered: *P*_*χ*_ = .99
Male [neutered, %]	12 (34.3%) [6, 50.0%]	24 (48%) [9, 37.5%]	
Weight (kg), n = 73	11.35 (2.3‐38.7)	16.15 (2.9‐50.0)	*P*_M_ = .08
Serum biochemistry			
Creatinine (mg/dL)	1.1 (0.6‐4.8)	1.2 (0.5‐6.3)	*P*_M_ = .29
Urea (mg/dL)	9.0 (2.4‐50.6)	10.8 (2.5‐45.7)	*P*_M_ = .56
Albumin (g/dL)	2.7 (0.5‐3.7)	2.35 (0.80‐3.50)	*P*_M_ = .06
Cholesterol, n = 84	338.8 (134.0‐660.9)	313.6 (83.1‐1397.9)	*P*_M_ = .5
UPC	6.20 (2.32‐20.30)	6.88 (2.04‐30.50)	*P*_M_ = .46
Severe proteinuria	26 (74%)	42 (84%)	*P*_*χ*_ = .41
Hypertension (n = 46)	9 (26%)	15 (30%)	*P*_*χ*_ = .99
IRIS CKD stage			
I	23 (66%)	28 (56%)	*P*_*χ*_ = .59
II	9 (26%)	15 (30%)	
III	3 (9%)	5 (10%)	
IV	0	2 (4%)	
Co‐morbidities			
Neoplasia	3 (9%)	7 (13%)	*P*_*χ*_ = .97
Hyperadrenocorticism	3 (9%)	5 (9%)	*P*_*χ*_ = .59
Diabetes mellitus	2 (6%)	4 (7%)	*P*_*χ*_ = .69
ACEi used			
Benazepril	33 (94%)	46 (92%)	*P*_*χ*_ = .69
Enalapril	2 (6%)	4 (8%)	
Starting dose of ACEi (mg/kg/day), n = 73	0.44 (0.16‐1.27)	0.50 (0.24‐1.52)	*P*_M_ = .14
Starting dose of ACEi <0.5 mg/kg/day	17 (49%)	19 (38%)	*P*_*χ*_ = .54
Renal support therapies given (started either before or at the time of ACEi)			
Renal diet	14 (40%)	18 (36%)	*P*_*χ*_ = .71
Omega‐3 supplementation	1 (3%)	7 (14%)	*P*_*χ*_ = .08
Aspirin	5 (14%)	10 (20%)	*P*_*χ*_ = .5
Clopidogrel	6 (17%)	14 (28%)	*P*_*χ*_ = .25

*Note*: Treatment response was defined as achieving a UPC of <0.5 or reduction of ≥50% from baseline within 3 months. Data presented as median (range) or n (% of population) as appropriate. Data were available for entire study cohort (n = 85) unless otherwise indicated.

Abbreviations: ACEi, angiotensin converting enzyme inhibitor; CKD, chronic kidney disease; *P*_M_, *P*‐value from a Mann‐Whitney statistical test; *P*_*χ*_, *P*‐value from a *χ* ^2^ statistical test; UPC, urine protein‐creatinine ratio.

Of the fifty dogs (59%) that were classified as non‐responders; 78% (n = 39/50) did not reach either UPC target within the first 3 months, 10% (n = 5/50) had their ACEi therapy withdrawn because of progressive azotemia, 8% (n = 4/50) were started on corticosteroid therapy and 4% (n = 2/50) were started on an ARB before either UPC target was reached. The median number of rechecks performed within 3 months of starting ACEi therapy was 1 (range, 1‐6). The median maximum dose for dogs that failed to reach UPC targets was 0.55 mg/kg/day (range, 0.24‐2.38 mg/kg/day).

One dog that responded to treatment died within 3 months of starting ACEi and 8 dogs that were classed as non‐responders died within this time frame.

Neither the presence of baseline azotemia (*χ* ^2^_1_ = 0.810, *P* = .37), hypoalbuminemia (*χ* ^2^_1_ = 1.83, *P* = .18), hypercholesterolemia (*χ* ^2^_1_ = 0.498, *P* = .48), severe proteinuria (*χ* ^2^_1_ = 0.976, *P* = .32) nor hypertension (*χ* ^2^_1_ = 0.056, *P* = .81) were found to be significantly associated with treatment response.

### 3.4 Outcome

#### 3.4.1 Association between treatment response and survival

**FIGURE 1:**
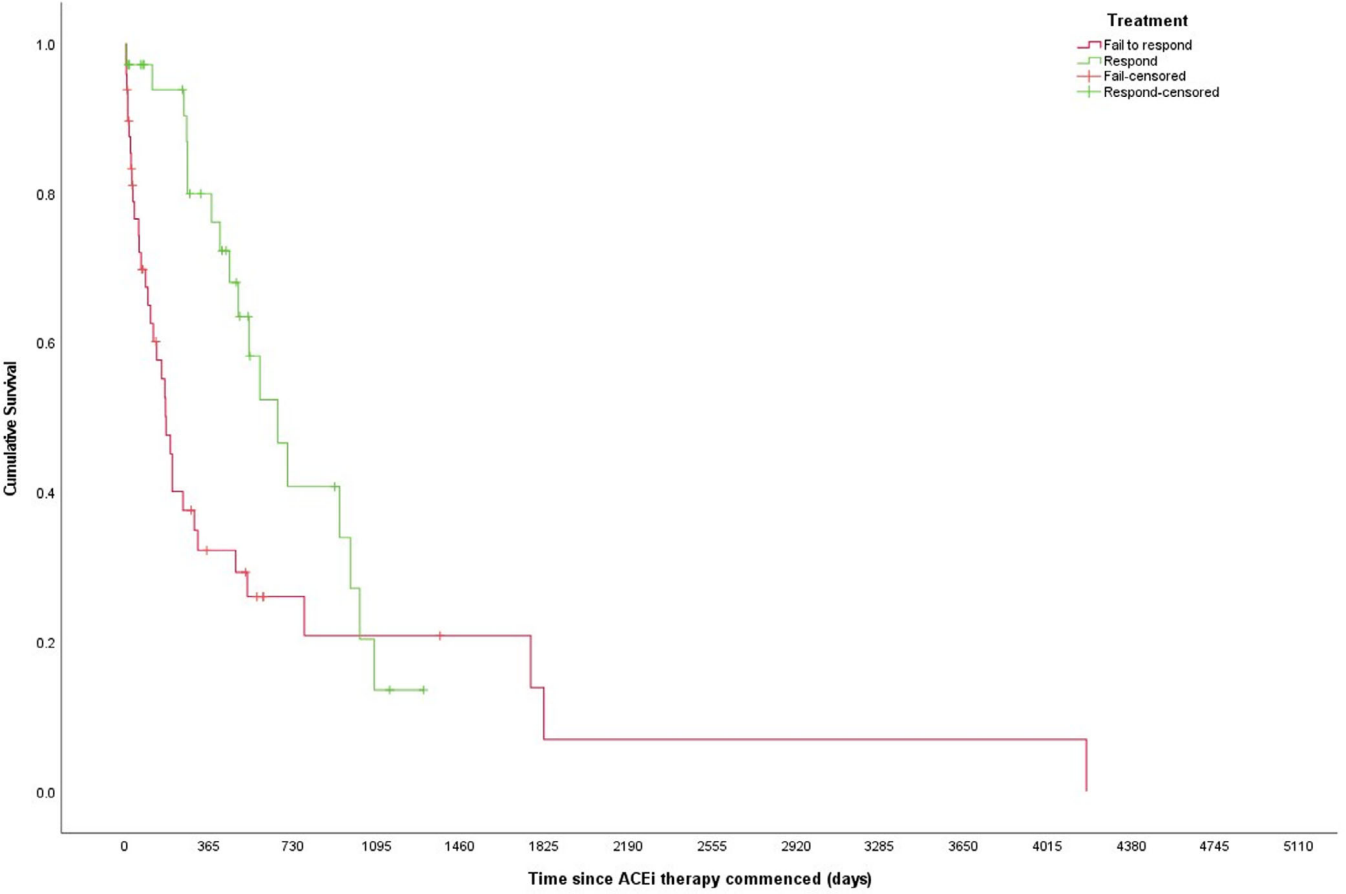
Kaplan‐Meier survival curve of all‐cause mortality for a cohort of 85 dogs with a urine protein‐creatinine ratio (UPC) > 2.0 that respond to or fail to respond to treatment with angiotensin converting enzyme inhibition. Treatment response was defined as achieving a UPC of <0.5 or reduction of ≥50% from baseline within 3 months of starting treatment. (Log‐rank Mantel Cox *P* = .009.)

There was a statistically significant difference in MST between responders and non‐responders (664 [95% CI: 459‐869] vs 177 [95% CI: 131‐223] days, respectively, *P* = .009; Figure 1). Of the 68 dogs with a known outcome at 12 months, 41% (n = 28/68) were responders. Of these, 79% (n = 22/28) were alive at 12 months while 28% (n = 11/40) of non‐responders were alive at 12 months; this difference was statistically significant (*χ* ^2^_1_ = 17.2, *P* < .001), with the odds of death greatly reduced (0.1) in responders (Table 2).

**TABLE 2:** Odds ratios from the univariable analysis of the association between response to treatment with an angiotensin converting enzyme inhibitor and baseline clinicopathological parameters and death by 12 months in a cohort of 85 dogs with a UPC >2.0.

Variable	OR	OR CI	*P* value
Either UPC target achieved within 3 months	0.10	0.03‐0.31	**<.001**
Baseline creatinine			
Values (mg/dL)	2.53	1.38‐5.61	**.008**
Azotemic	5.34	1.85‐17.32	**.003**
Baseline albumin			
Values (g/dL)	0.40	0.18‐0.82	**.02**
Hypoalbuminemic	4.51	1.66‐13.14	**.004**
Baseline cholesterol			
Values (mg/dL)	1.00	1.00‐1.00	.74
Hypercholesterolemia	0.95	0.36‐2.51	.92
Baseline urine protein‐creatinine ratio (UPC)			
Values	1.18	1.05‐1.34	**.01**
Severe proteinuria (UPC > 3.5)	2.91	0.84‐11.79	.11
Baseline systolic blood pressure			
Hypertensive (systolic blood pressure ≥ 160 mm Hg)	2.36	0.65‐9.05	.2

*Note*: Treatment response was defined as achieving a UPC of <0.5 or reduction of ≥50% from baseline within 3 months of starting treatment. Bold values represent statistically significance.

Abbreviations: OR, odds ratio; OR CI, odds ratio 95% confidence interval; *P* value, associated statistical significance; UPC, urine protein‐creatinine ratio.

#### 3.4.2 Association between clinicopathological variables and hypertension on survival

**FIGURE 2:**
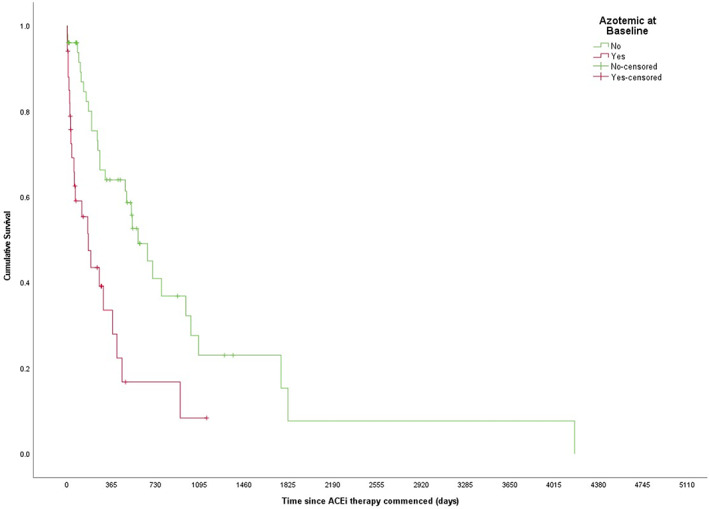
Kaplan‐Meier survival curve of all‐cause mortality for a cohort of 85 dogs with a urine protein‐creatinine ratio > 2.0 with and without baseline azotemia. (Log‐rank Mantel Cox *P*≤ .001.)

The MST of dogs that were or were not azotemic at the time of diagnosis also differed significantly (175 [95% CI: 62‐288] vs 586 [95% CI: 398‐773] days, respectively), *P* < .001; Figure 2). While dogs that were hypoalbuminaemic at baseline had a shorter MST than those that were not (177 [95% CI: 115‐239] vs 531 [95% CI: 373‐689] days, respectively), this was not statistically significant (*P* = .06). There was no statistically significant difference in the MST for dogs that had hypercholesterolemia at baseline compared to those that did not (375 [95% CI: 28‐722] vs 412 [95% CI: 145‐679] days, respectively, *P* = .24) nor those with severe proteinuria compared to those that did not (300 [95% CI: 63‐537] vs 531 [95% CI: 403‐659] days, respectively, *P* = .92) or hypertension (hypertensive—251 [95% CI: 0 = 532] vs normotensive—454 [95% CI: 240 = 668] days, *P* = .37).

**FIGURE 3:**
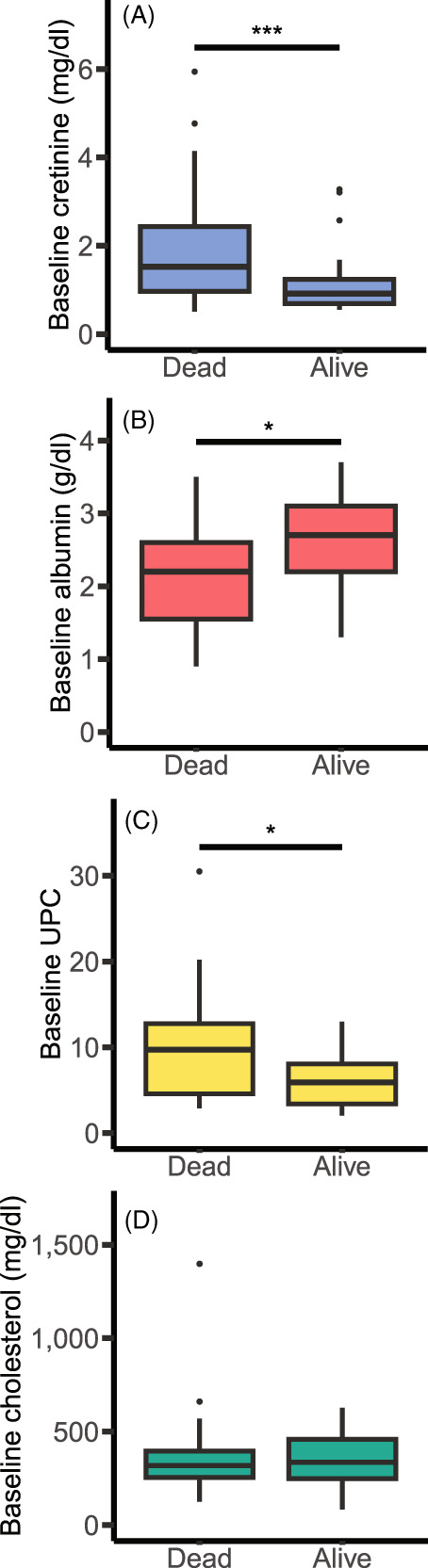
Box and Whisker Plots for clinicopathological variables at baseline and survival status at 12 months for dogs with a urine protein‐creatinine ratio (UPC) > 2.0 treated with angiotensin converting enzyme inhibition. 12‐month survival status was known for 68 dogs. (A) Creatinine (mg/dL); (B) Albumin (g/dL); (C) UPC, and (D) Cholesterol (mg/dL). ● Indicate values >1.5× interquartile range. Mann‐Whitney *U* test was performed. Statistical significance indicated by ***<.001; *<.05.

When assessed as continuous variables, creatinine, albumin, and UPC were found to be statistically significantly associated with 12‐month outcome while cholesterol was not (Figure 3A‐D). Furthermore, logistic regression estimated 2.53 and 1.18 increases in the odds of death with each 1.1 mg/dL (100 μmol/L) increase in creatinine and each unit increase in UPC, respectively, a reduction in odds (0.40) with each 0.1 g/dL increase in albumin and no change in odds associated with increasing cholesterol (1.00; Table 2). Qualitatively similar results were obtained when creatinine, albumin, cholesterol, UPC and SBP were assessed in terms of clinical thresholds. The presence of baseline azotemia (*χ* ^2^_1_ = 9.5, *P* = .003, OR = 5.34; Table 2) and hypoalbuminemia (*χ* ^2^_1_ = 8.7, *P* = .004, OR = 4.51) were both negatively associated with 12‐month survival, whereas there was no difference in survival at 12 months in dogs with or without hypercholesterolemia (n = 17/36; *χ* ^2^_1_ < 0.01, *P* = .92, OR = 0.95) or those with or without severe proteinuria (*χ* ^2^_1_ = 2.756, *P* = .11, OR 2.91). Twelve‐month survival data were known for 38 dogs with baseline SBP readings available; 60% (n = 12/20) of the dogs that were not hypertensive were alive at 12 months compared to 39% (n = 7/18) of hypertensive dogs. Hypertension at baseline was not associated with 12‐month survival (*χ* ^2^_1_ = 1.689, *P* = .2, OR = 2.36).

To remove any impact of baseline azotemia and hypoalbuminemia on outcome, 12‐month survival analysis was repeated on the sub‐group of 25 dogs without these abnormalities present. Response to treatment remained significantly associated with survival at 12 months in this cohort of dogs (*χ* ^2^_1_ = 4.425, *P* = .04, OR = 0.01).

### 3.5 Reasons for euthanasia

Thirty‐five dogs were known to be dead at the 12‐month follow‐up date. Of these dogs, reason for death was known for 24 (69%). Progression or presence of renal disease was cited as at least part of the reason for euthanasia in 88% of cases (n = 21/24). Reasons for euthanasia in the remaining dogs were progression of neoplasia (n = 1), development of cardiorespiratory disease (n = 1), and progression of lymphoma along with respiratory compromise (n = 1).

### 3.6 Multivariable analysis

Multivariable logistic regression analysis to evaluate the relative importance of azotemia or hypoalbuminemia given treatment response found that even considering the reduction in odds of death in responders, the presence of both azotemia and hypoalbuminemia at baseline were still associated with increased odds of death at 12 months (7.69 and 4.66, respectively; Table 3).

**TABLE 3:** Factors found on multivariable analysis to be significantly associated with death by 12 months in a cohort of 85 dogs with a UPC > 2.0 treated with ACEi.

Predictors	OR	OR CI	*P* value
Either UPC target achieved within 3 months	0.10	0.02‐0.34	.001
Azotemic at baseline	7.69	1.95‐39.55	.007
Hypoalbuminemic at baseline	4.66	1.32‐19.04	.02

*Note*: Treatment response was defined as achieving a UPC of <0.5 or reduction of ≥50% from baseline within 3 months of starting treatment.

Abbreviations: OR, odds ratio; OR CI, odds ratio 95% confidence interval; *P* value, associated statistical significance; UPC, urine protein‐creatinine ratio.

### 3.7 Categorical tree

**FIGURE 4:**
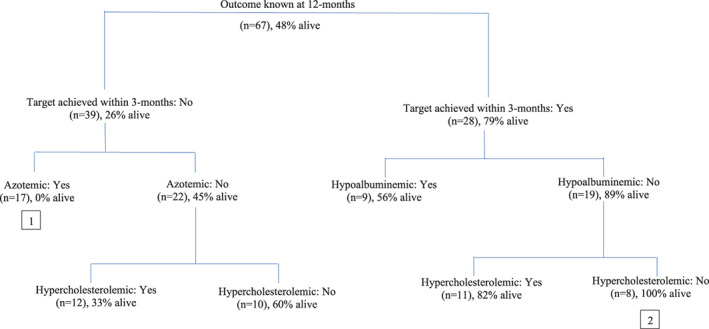
Categorical tree showing effect of achieving either urine protein‐creatinine ratio (UPC) target within 3 months and the presence of baseline biochemical abnormalities on survival at 12 months in a cohort of dogs with a UPC > 2.0 treated with angiotensin converting enzyme inhibition. Treatment response was defined as achieving a UPC of <0.5 or reduction of ≥50% from baseline within 3 months of starting treatment. (n) = number of dogs with that combination of factors; % = % of that combination known to be alive at 12 months. (1 & 2) in boxes see results text.

Finally, to evaluate the interplay between the presence of a combination of factors in terms of clustering of clinical thresholds and response to treatment in particular dogs, a categorical tree was generated. Although 12‐month survival data were available for 68 dogs, only 67 dogs were included in the categorical tree as cholesterol was unavailable for 1 dog. Hypertension was not included in this model as SBP was not available for all dogs. The categorical tree determined the principal factor influencing 12‐month survival to be treatment response. The worst outcome was seen in dogs that failed to respond to ACEi within 3 months and that were azotemic; none of these dogs (n = 17) were alive at 12 months (Figure 4, [1]). Conversely, the best outcome was seen in dogs that responded to treatment and which had normal albumin and cholesterol at baseline; all dogs (n = 8) with this combination of factors were alive at 12 months (Figure 4, [2]).

## 4 DISCUSSION

We set out to evaluate the frequency that ACEi treatment resulted in a ≥50% reduction in UPC or a UPC < 0.5 within 3 months in a cohort of dogs with a UPC > 2.0, and to evaluate whether achieving this target conveyed survival benefit. Although only 41% of our study cohort achieved 1 of the targets for reduction in UPC within 3 months, those that did were significantly more likely to be alive 12 months after starting treatment with responders also having significantly longer MST. The presence of baseline azotemia or hypoalbuminemia, and magnitude of proteinuria, were also associated with a worse outcome, although to a lesser extent.

There are numerous studies in the human literature supporting the use of ACEi in proteinuric renal diseases, including diabetic nephropathy,^13^, ^14^ non‐diabetic nephropathy,^15^, ^16^, ^17^, ^18^ and IgA nephropathy,^19^, ^20^ to slow the progression to end‐stage renal failure. Similarly, there is evidence within veterinary literature to support their use in the management of proteinuric renal disease. Enalapril reduces proteinuria and slows disease progression in some dogs with idiopathic glomerulonephritis^21^ and delays the increase in serum creatinine and UPC in dogs with X‐linked hereditary nephritis.^22^ What is less clear is whether the benefits of ACEi are associated with the magnitude of reduction in proteinuria. While ACEi reduce proteinuria via their effect on glomerular hemodynamics,^23^, ^24^, ^25^ they also lead to a decrease in the production of vasoactive substances implicated in development of glomerulosclerosis, delay the growth and proliferation of mesangial cells, and reduce the degradation of bradykinin, all of which could influence the rate of disease progression independent of the magnitude of reduction of proteinuria.^24^, ^26^, ^27^, ^28^, ^29^, ^30^ Given that dogs achieving a 50% reduction in UPC were 10 times more likely to be alive at 12 months compared to those that did not and had a significantly longer MST, it appears that outcome is associated with the magnitude of reduction in UPC in response to ACEi. This strong association supports the currently recommended target of >50% reduction in UPC, justifying both the time and financial commitments needed to achieve this target.

Treatment response was not the only variable found to be associated with survival. The presence of baseline azotemia, hypoalbuminemia, and magnitude of baseline creatinine, albumin, and UPC were all negatively associated with 12‐month survival. This is unsurprising as increasing IRIS stage, the presence of nephrotic syndrome and increased UPC are negative prognostic indicators in a variety of renal diseases of dogs.^12^, ^31^, ^32^, ^33^, ^34^ Similarly, in human IgA nephropathy, baseline creatinine and magnitude of proteinuria are independent predictors of progression to end‐stage renal disease.^35^

While multivariable analysis confirmed that azotemia and hypoalbuminemia were independent risk factors for death before 12 months, response to therapy remained positively associated with 12‐month survival in the subset of dogs with less severe disease (ie, with normal creatinine and albumin). Additionally, the categorical tree analysis provided a clear visual representation of the association between the categorical variables assessed and 12‐month survival. Although the presence of azotemia had a negative association and lack of hypoalbuminemia a positive association, treatment response ranked highest in its association with survival at 12 months. Therefore, although the presence of markers of disease severity (azotemia and hypoalbuminemia) appears to be negative prognostic indicators, response to treatment is suggested to have the strongest association with 12‐month survival and hence these results again support the currently recommended target of a 50% reduction in UPC regardless of disease severity.

When UPC was assessed as a continuous variable it was found to be associated with 12‐month survival, however, when it was assessed as a categorical variable the same significance was not found. Severe proteinuria was defined as a UPC > 3.5; as per the ACVIM Consensus Guidelines.^10^ Eight percent of dogs had severe proteinuria and hence it possible that this study was underpowered to detect a difference between the 2 groups. The cut‐off of 3.5 to define severe proteinuria might also be too low in cases of glomerular protein loss; additional studies are required to further interrogate this cut‐off.

As reported in people with proteinuric renal disease^36^ and in previous studies of dogs,^21^, ^37^ a variable response to ACEi was observed in our study cohort, with only about 40% of dogs reaching the target UPC. This is unsurprising as ACEi therapy does not address the underlying cause of glomerular disease and treatment with ACEi is unlikely to result in complete resolution of glomerular injury.^6^ Additionally, in our study, inadequate dose escalation could have been a factor in failure to achieve response. Other potential explanations for this inconsistent response include angiotensin converting enzyme (ACE) gene polymorphisms,^38^, ^39^, ^40^, ^41^ differing etiology for proteinuria,^42^ and disease severity or magnitude of the proteinuria at time of starting ACEi therapy.^43^ Lack of response to ACEi could also occur because of incomplete suppression of angiotensin II synthesis either via incomplete inhibition of ACE or production via ACE‐independent pathways.^44^ Aldosterone breakthrough (increased aldosterone levels despite ACEi treatment) occurs in a subset of human patients.^45^, ^46^, ^47^ While this phenomenon has not been widely investigated in dogs, 34%‐59% of dogs with proteinuric CKD receiving RAAS inhibitors demonstrate aldosterone breakthrough; as do 32% of dogs treated with benazepril for cardiac disease.^48^, ^49^ Aldosterone breakthrough might also account for the subset of dogs that initially responded to treatment but had subsequent increases in UPC that did not meet either UPC target. The variability in response to ACEi and the clear survival benefit associated with achieving target reductions in UPC, support the recent focus on alternative or adjunctive methods of RAAS suppression when ACEi are insufficient.

In humans, combination therapy of an ACEi and ARB is reported to have a possible synergistic effect with a recent meta‐analysis review suggesting combination therapy to be both safe and effective.^50^, ^51^ Combination therapy has not yet been widely studied in veterinary medicine, however, ARBs, specifically telmisartan, are becoming more frequently prescribed and represent an alternative to ACEi treatment. While literature on the use of telmisartan in dogs is sparse, promising data is now emerging. In a cohort of dogs with a UPC of ≥2.0 (if non‐azotemic) or ≥0.5 (if azotemic) 3 months after telmisartan treatment 68% had reached target UPC (defined as per the current study) while a UPC of <0.5 was achieved in 9%‐14% of dogs.^52^ A UPC < 0.5 was also achieved in 21% of dogs treated with telmisartan for proteinuric CKD.^53^ The use of telmisartan was directly compared to enalapril with a change in UPC from baseline to day 30 significantly greater in dogs with proteinuric CKD (UPC > 0.5 and azotemic or ≥1.0 if non‐azotemic) treated with telmisartan.^37^

Response to ACEi in this study was not associated with disease severity as defined by the presence of azotemia or hypoalbuminemia. In the human literature, the use of ACEi slows progression of disease in patients with severe renal failure.^54^ Our results suggest that, similarly to human medicine, dogs that are azotemic at the time of starting ACEi might still show a positive response and benefit from improved survival. This has possible clinical relevance as there is often hesitation to start ACEi therapy in dogs with pre‐existing azotemia because of concerns that ACEi‐associated alterations in renal hemodynamics will lead to worsening of azotemia. Response to ACEi was not found to be associated with the presence of baseline hypertension. When the human literature is reviewed this finding is perhaps not surprising with several studies showing ACEi to have beneficial effects that are only in part because of the reduction in blood pressure.^15^, ^16^, ^36^

This study had several important limitations, including the decision to use a UPC of >2.0 as an inclusion criterion to recruit cases with presumed glomerular protein loss. While UPC of >2.0 has been widely accepted as an indicator of glomerular proteinuria,^1^ several more recent studies have reported UPC > 2.0 in dogs with histopathologically confirmed tubulointerstitial disease^25^, ^55^ and therefore it is possible that some included dogs did not have primary glomerular disease. Similarly, dogs with confirmed glomerular disease can have a UPC < 2.0^56^ and would have been excluded from our study, again potentially influencing our conclusions. Given the retrospective nature of this study, with case enrollment starting from 2006, the presence of glomerulonephropathy could not be definitively confirmed or characterized.

Due to the retrospective nature of this study, dogs did not undergo a standardized work‐up and comprehensive screening for potential triggers was not performed in all cases. It is also possible that some dogs could have had pre‐ or post‐renal conditions contributing to their proteinuria. Additionally, the starting dose of ACEi and timing of dose escalations was uncontrolled; a substantial proportion of dogs were started on a dose that was below the recommended starting dose and few dogs had their dose of ACEi increased to the upper limits of the dose range. The median dose at the time of response was 0.52 mg/kg/day which is the current recommended starting dose^16^ and median time to response was 33 days. The median maximum dose for dogs that failed to reach UPC targets was 0.55 mg/kg/day (range, 0.24‐2.38 mg/kg/day). This may account for the low proportion of dogs reaching UPC targets and it is possible that the numbers of responders would have been higher if dogs had been treated more aggressively and further dose escalations pursued. Dose escalation of ACEi improves response in dogs,^37^ although not always.^21^ The median starting UPC in the study of dogs that reported an improvement with dose escalation was 2.29, whereas in our study it was 6.61, and this might have increased the likelihood of achieving a treatment response.^37^ In human medicine, ACEi dose optimization improves outcome.^57^, ^58^ The cohort of dogs in this study was heterogenous, particularly with respect to their concurrent diagnoses; numerous dogs had a co‐morbidity that could have increased their UPC and if such co‐morbidities were not well controlled then it is possible that regardless of the efficacy of the ACEi therapy the UPC might have remained abnormally high. Conversely, treatment of underlying diseases could decrease proteinuria independently of ACEi therapy. The study cohort was chosen to reflect the situation commonly seen in clinical practice, where standard therapies (eg, ACEi) for glomerular diseases are often started without performing renal biopsies and in the presence of co‐morbidities and whereas, the presence of co‐morbidities could also have affected the reliability of survival data, over 85% of the dogs with known cause of death had progression of renal disease listed as at least a contributing factor to the reason behind their euthanasia, supporting this decision.

Concurrent therapy for the treatment of glomerular proteinuria or its complications was also not controlled. Some enrolled dogs were already receiving a prescription renal diet whereas others were started on a renal diet at the time of starting ACEi therapy and others were never fed such a diet. Feeding a renal diet has previously been shown to reduce UPC in dogs with proteinuric CKD.^59^ Omega‐3 supplementation, antithrombotic and anti‐hypertensive therapy were also not controlled. Whether diet and the aforementioned additional therapies contributed to decreasing UPC and/or improving survival in this study is unknown.

In conclusion, this study suggests response to treatment with ACEi in dogs with a UPC > 2.0 conveys a significant survival benefit, however, achieving target UPCs was only achieved in 40% of dogs on treatment with ACEi. The presence of baseline azotemia, hypoalbuminemia and the magnitude of proteinuria were found to be independent negative prognostic indicators in these dogs.

## CONFLICT OF INTEREST DECLARATION

Authors declare no conflict of interest.

## OFF‐LABEL ANTIMICROBIAL DECLARATION

Authors declare no off‐label use of antimicrobials.

## INSTITUTIONAL ANIMAL CARE AND USE COMMITTEE (IACUC) OR OTHER APPROVAL DECLARATION

Approved by University of Glasgow, ethical approval reference EA22/21.

## HUMAN ETHICS APPROVAL DECLARATION

Authors declare human ethics approval was not needed for this study.
